# To Render Ivory Flexible

**Published:** 1881-02

**Authors:** 


### TO RENDER IVORY FLEXIBLE.

Ivory is readily rendered quite flexible by immersion in a solution of pure phosphoric acid (specific gravity 1·13) until it loses, or partially loses, its opacity, when it is washed in clean cold water and dried. In this state it is as flexible as leather, but gradually hardens by exposure to dry air. Immersion in hot water, however, restores its softness and pliancy. The following method may also be employed: Put the ivory to soak in three ounces nitric acid mixed with fifteen ounces water. In three or four days the ivory will be soft.

